# Bone-Derived Modulators That Regulate Brain Function: Emerging Therapeutic Targets for Neurological Disorders

**DOI:** 10.3389/fcell.2021.683457

**Published:** 2021-06-10

**Authors:** Hongzhen Chen, Dewei Shang, Yuguan Wen, Chao Liang

**Affiliations:** ^1^Department of Biology, Southern University of Science and Technology, Shenzhen, China; ^2^Department of Pharmacy, The Affiliated Brain Hospital of Guangzhou Medical University, Guangzhou, China

**Keywords:** bone, bone-derived proteins, bone marrow-derived cells, brain function, neurological disorders

## Abstract

Bone has traditionally been regarded as a structural organ that supports and protects the various organs of the body. Recent studies suggest that bone also acts as an endocrine organ to regulate whole-body metabolism. Particularly, homeostasis of the bone is shown to be necessary for brain development and function. Abnormal bone metabolism is associated with the onset and progression of neurological disorders. Recently, multiple bone-derived modulators have been shown to participate in brain function and neurological disorders, including osteocalcin, lipocalin 2, and osteopontin, as have bone marrow-derived cells such as mesenchymal stem cells, hematopoietic stem cells, and microglia-like cells. This review summarizes current findings regarding the roles of these bone-derived modulators in the brain, and also follows their involvement in the pathogenesis of neurological disorders. The content of this review may aide in the development of promising therapeutic strategies for neurological disorders via targeting bone.

## Introduction

The brain is a complex and powerful organ consisting of a great variety of nerve tissues that plays essential roles in coordinating body homeostasis (Qin et al., 2016). The brain also directly and indirectly regulates almost all body behaviors, including executive and cognitive functions, immunity, reproduction, and glucose metabolism, through neuroendocrine signaling (Elenkov et al., 2000; Kane and Engle, 2002; Kauffman et al., 2007; Jia et al., 2020). Meanwhile, the brain is influenced by the feedback effects from other organs. A famous example is the microbiota-gut-brain axis (Vuotto et al., 2020). Gut microbes can affect brain functions by multiple mechanisms, such as neural, immunoregulation, and endocrine pathways. Indeed, disturbances in the intestinal microbiota balance contribute to the onset and progression of central nervous system diseases (Logsdon et al., 2018).

Bone is a hard tissue viewed as a scaffold to support and protect the various organs of the body and, historically, has been regarded as an “independent” organ (Buckwalter et al., 1996). However, in recent years, bone has proven to be an endocrine organ. Osteocalcin (OCN), a hormone secreted by osteoblasts, was reported to take part in systemic body regulation by acting on adipose tissue, muscle, pancreas, and male gonads (Wei et al., 2014; Kover et al., 2015; Sabek et al., 2015; De Toni et al., 2016a,b; Gao J. et al., 2016; Khrimian et al., 2017; Cristina Diaz-Franco et al., 2019).

Although bone and brain tissue seem apparently unrelated, both clinical and experimental research have proposed a bilateral dependence of the two organs (Otto et al., 2020). A large number of well-established studies indicate that the brain can influence bone metabolism by several pathways, including (a) secretion of hypothalamic peptides that directly regulate bone, (b) transduction of neuronal signals that act on bone through the sympathetic nervous system, and (c) transduction of hypothalamic neuroendocrine signals that act on bone via the pituitary (Quiros-Gonzalez and Yadav, 2014; Dimitri and Rosen, 2017). However, the roles of bone-derived modulators in the regulation of brain development and function are often underestimated.

Increasing studies have shown that bone can exert regulation on the brain by secreting various molecules, several of which are essential to brain homeostasis (Chamouni et al., 2015). Abnormal bone metabolism may also contribute to the occurrence and development of neurodegenerative and neuropsychiatric disorders (Downey et al., 2017; Yuan et al., 2019). Patients with neurological disorders, the second main cause of death worldwide in 2016, still have no access to effective treatments (Feigin et al., 2019). These unexpected and remarkable discoveries provide a new perspective to explore how bone regulates brain function, and an opportunity to develop promising therapeutic methods for neurological disorders via targeting bone.

The purpose of this review is to provide a detailed overview of the bone-derived modulators that have the capability to regulate brain functions, and to summarize the current knowledge about the relevance of the bone-derived modulators in the pathogenesis of neurological disorders.

## Impact of Bone-Derived Modulators on the Brain

**FIGURE 1:**
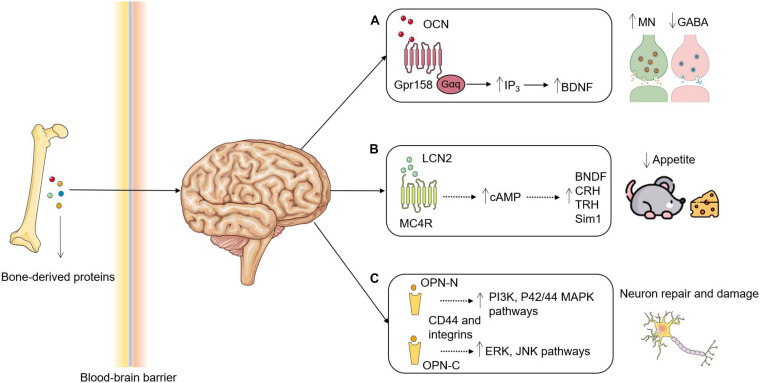
Roles of bone-derived proteins in brain functions. **(A)** OCN is decarboxylated and secreted by osteoblasts and enters the circulation to act as an endocrine hormone. OCN transverses the BBB, where it binds to Gpr158 in the brain to increase the synthesis of BDNF through upregulating IP3 and eventually results in promoting MN synthesis and inhibiting GABA production. **(B)** LCN2 is secreted by osteoblasts and binds to MC4R in the hypothalamus after crossing the BBB. Activation of MC4R by LCN2 induces cAMP and promotes the synthesis of BDNF, CRH, TRH and Sim1 to suppress appetite. **(C)** The proteases cleavage may generate two OPN fragments: OPN-N and OPN-C. OPN-N binds with integrins to activate PI3K and P42/44 MAPK pathways leading to a protective role, whereas OPN-C binds with CD44 to activate ERK and JNK pathways leading to a detrimental role. OCN, Osteocalcin; BBB, blood-brain barrier; Gpr158, G protein-coupled receptor 158; BDNF, brain-derived neurotrophic factor; IP3, inositol triphosphate; MN, monoamine neurotransmitters; Gαq, guanine nucleotide-binding protein α-q; LCN2, lipocalin-2; MC4R, melanocortin 4 receptor; cAMP, cyclic adenosine monophosphate; CRH, corticotropin releasing hormone; TRH, thyrotropin-releasing hormone; Sim1, single-minded homolog 1; OPN, osteopontin. OPN-N/C, N/C-terminal fragment of OPN. Figures were produced using Servier Medical Art, modified.

Bone mainly regulates brain development, function, and pathophysiology by secreting various proteins, including OCN, lipocalin-2 (LCN2), and osteopontin (OPN) (Figure 1), and providing cells such as bone-derived mesenchymal stem cells (BMSCs), hematopoietic stem cells, and microglia-like cells (Otto et al., 2020).

### OCN

OCN, also known as bone gamma-carboxyglutamate protein, is exclusively secreted by osteoblasts and decarboxylated by osteoclasts into undercarboxylated OCN, which is an active molecule acting as an endocrine hormone (Price et al., 1976; Mizokami et al., 2017). In the peripheral, OCN binds to the G protein-coupled receptor family C group 6 member A (Gprc6a) to exert endocrine functions, including regulating insulin secretion and testosterone production, and promoting muscle adaptation to exercise (Mizokami et al., 2017). OCN was discovered to transverse the blood-brain barrier (BBB), inhibit γ-aminobutyric acid (GABA) synthesis, and enhance monoamine neurotransmitters synthesis by binding specifically to neurons in the hippocampus, brainstem, and midbrain (Oury et al., 2013; Khrimian et al., 2017). G protein-coupled receptor 158 (Gpr158) was the first receptor that was discovered in the brain for OCN and is expressed in the somatosensory, motor, and auditory areas of the cortex, and in the piriform cortex, the retro-splenial area, and the ventral tegmental area. Behavioral experiments discovered that, compared with wild-type littermates, adult mice lacking OCN exhibited a substantial increase in anxiety-like behavior and had a major deficit in memory and learning (Nakazawa et al., 2002; Oury et al., 2013). Administration of OCN to the OCN-lacking mice reduced anxiety-like behavior and improved their memory and learning capabilities (Villeda et al., 2014). Moreover, maternal OCN was required for the fetal brain to regulate the progression of neurogenesis and prevent neuronal apoptosis, and it was necessary for optimal memory and spatial in the adult offspring (Oury et al., 2013).

### LCN2

Another bone-derived hormone, LCN2, also known as 24p3 and neutrophil gelatinase-associated lipocalin, is an osteoblast-enriched glycoprotein (Borregaard and Cowland, 2006). LCN2 was previously thought to be exclusively secreted by adipose tissue, but increasing evidence suggests that bone is the predominant organ expressing LCN2, with tenfold or higher expression levels compared with adipose tissue (Soukas et al., 2000; Yan et al., 2007; Mosialou et al., 2017). Similar to OCN, LCN2 was discovered to participate in energy metabolism by regulating insulin secretion and improving glucose tolerance and insulin sensitivity (Yan et al., 2007). Recently, LCN2 was found to activate the anorexigenic pathway by binding to the melanocortin 4 receptor in the paraventricular nucleus and ventromedial neurons of the hypothalamus after crossing the BBB (Ste Marie et al., 2000; Liu et al., 2003; Mosialou et al., 2017). Furthermore, studies reported that LCN2 was involved in direct neurotoxic effects by interacting with the LCN2 receptor on neurons and increasing the sensitivity of neurons to cell death evoked by oxidative stress, nitric oxide, and tumor necrosis factor (Devireddy et al., 2005; Tong et al., 2005; Nelson et al., 2008; Mucha et al., 2011). LCN2 also enhanced neuronal motility and inflammatory responses through activation of the JAK2, STAT3 and NF-kB pathways to upregulate expression of C-X-C motif chemokine 10 (Yan et al., 2007; Bauer et al., 2008; Lee et al., 2012). These findings indicate that LCN2 can influence the development of the central nervous system and its neuropathology by regulating neuronal cell death, migration, and morphology.

### OPN

OPN is a secreted matricellular protein that was originally discovered in bone and was later shown to be expressed in various tissues including kidney, epithelial linings, skeletal muscle, mammary, and brain (Franzen and Heinegard, 1985; Yokosaki et al., 2005; Clemente et al., 2016). OPN was reported to play important roles in tissue remodeling, immune regulation, and biomineralization by binding to multiple receptors, including various integrins (αvβ1, αvβ3, αvβ5, αvβ6, α4β1, α5β1, α8β1, and α9β1) and CD44 (Lin and Yang-Yen, 2001; Yokosaki et al., 2005). In bone, OPN was thought to promote bone resorption by anchoring osteoclasts to the mineral matrix of bones (Reinholt et al., 1990; Yoshitake et al., 1999; Ishijima et al., 2001). Patients with high serum OPN concentrations had low bone mineral density (Cho et al., 2013; Filardi et al., 2019). Interestingly, OPN was discovered to exert conflicting functions in the brain. Several studies demonstrated that OPN protected neurons and promoted repair in acute brain injuries and neurodegenerative diseases by coordinating inflammatory responses, anti-apoptotic actions, BBB maintenance, and chemotaxis and proliferation of nerve cells (Denhardt et al., 2001; Lin and Yang-Yen, 2001; Mazzali et al., 2002; Zhou et al., 2020). In contrast, other studies suggested that OPN served as a proinflammatory cytokine, recruiting harmful inflammatory cells to lesion sites and contributing to neurological diseases (Chabas et al., 2001; Maetzler et al., 2007; Rentsendorj et al., 2018). A possible explanation for the above conflicting findings is that OPN formed different fragments after proteases cleavage, which could bind to distinct receptors (CD44 and integrins) to activate different signaling pathways and cell responses (Boggio et al., 2016). OPN has been reported to activate P42/44 MAPK and PI3K pathways to exerts neuroprotective function, whereas activation of JNK and ERK pathways evokes detrimental neuroinflammation by upregulating the expression of proinflammatory cytokines (Meller et al., 2005; Wang et al., 2019).

### Bone Marrow-Derived Cells

**FIGURE 2:**
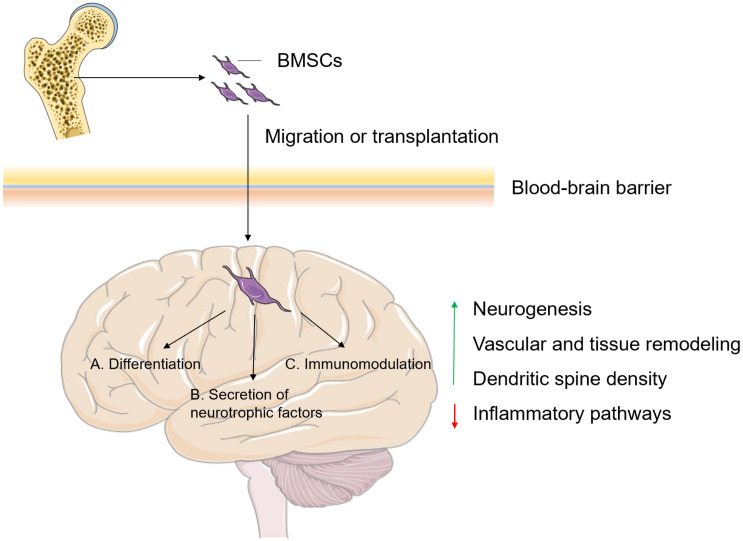
The possible mechanisms of BMSCs involved in the regulation of brain functions. BMSCs can be transplanted directly into the injury site, or they can actively migrate to the injured site after crossing the BBB, where **(A)** they differentiate into neuronal lineages and oligodendrocytes; **(B)** elicit nerve regeneration by secreting neurotrophic factors and **(C)** act as immunomodulatory cells, eventually leading to promoted neurogenesis, vascular and tissue remodeling, and dendritic spine density and inhibited inflammatory pathways. BMSCs, bone mass-derived mesenchymal stem cells; BBB, blood-brain barrier. Figures were produced using Servier Medical Art, modified.

Bone marrow, a tissue with a malleable and sponge-like texture, plays an active role in the body by producing various types of cells, such as hematopoietic stem cells, microglia-like cells and BMSCs. For hematopoietic stem cells, they have been demonstrated to act on the brain by secreting cytokines and hematopoietic growth factors, which can inhibit apoptosis, increase neurogenesis and also promote the migration of bone marrow-derived microglia-like cells into the brain (Sanchez-Ramos et al., 2008). Bone marrow-derived microglia-like cells have potential therapy against AD through phagocytic clearance of protein aggregates and cellular debris to maintain homeostasis in the brain (Arandjelovic and Ravichandran, 2015). Additionally, BMSCs are pluripotent stem cells and have been proved to migrate to areas afflicted by neurological insults or neurodegeneration (Borlongan et al., 2011). When compared with other types of bone marrow-derived cells, BMSCs have been widely studied because of their availability, ease of isolation and culture, powerful self-renewal, and multilineage differentiation (Grove et al., 2004; Badyra et al., 2020). The possible mechanism by which BMSCs act on brain tissue can be summarized as follows (Figure 2): (1) BMSCs could migrate to the injured brain areas via passing through the BBB with the help of several receptors, integrins, selectins and proteolytic enzymes. One of the critical chemoattractants for BMSCs migration is SDF-1 (stromal cell-derived factor-1). The level of SDF-1 was significantly increased under pathological conditions such as inflammation, ischemia or hypoxic. SDF-1 bound with CXCR4 to attracting the BMSCs to migrate into the damaged region. However, only minute quantities of bone BMSCs can migrate to the brain under non-pathological conditions (Lee et al., 2015; Bang et al., 2017).; (2) BMSCs have the potential to differentiate into neuronal lineages and oligodendrocytes by forming primary neurospheres (Balasubramanian et al., 2013); (3) BMSCs are regarded as immunomodulatory cells that could effectively inhibit the inflammatory state by regulating inflammatory signaling. BMSCs also directly influence inflammation through direct cell-cell contact and secretion of soluble factors (Di Trapani et al., 2013; Gao F. et al., 2016; Simon et al., 2017); and (4) BMSCs elicit nerve regeneration by secreting nerve growth factor, glial cell-line-derived neurotrophic factor, brain-derived neurotrophic factor, and vascular endothelial growth factor (Zhang et al., 2004; Uccelli et al., 2011). BMSCs therapy may be one of the most promising treatments for neurodegeneration, as this approach has been proven to be safe and has great potential to improve the symptoms of neurological diseases (Pernia et al., 2020). In the next part, we reviewed the research progress in the application of BMSCs in the treatment of neurological disorders.

## The Role of Bone-Derived Modulators in Neurodegenerative Diseases

Neurodegenerative diseases, a heterogeneous group of disorders, cause severe motor and cognitive impairments and are characterized by the relentlessly progressive degeneration of the functional and structural integrity of the central nervous system and peripheral nervous system (Heemels, 2016). Different neurodegenerative diseases may share the same neuropathological features, such as neuronal loss, neuroinflammation, BBB impairment, protein misfolding, and autophagy dysfunction (Menzies et al., 2015; Riek and Eisenberg, 2016; Wyss-Coray, 2016). Below, we review the roles of bone-derived modulators in neurodegenerative diseases, including Alzheimer’s disease (AD) and Parkinson’s disease (PD), and stroke (Table 1).

**TABLE 1:** Bone-derived modulators involved in neurological disorders.

Factors	Role in	Organism or model	Correlated observation	Potential action
OCN	PD	PD rats	Downregulated in CSF	Increase in dopamine synthesis; improvement in behavioral dysfunctions; reduction in the loss of tyrosine hydroxylase; improvement in astrocytic and microglial functions.
	MD	Patients with depression	Downregulated in plasma	Increase in serotonin, dopamine, and noradrenaline release and reduction in GABA release.
OPN	AD	Patients with AD	Upregulated in plasma and CSF especially in early stage AD	Protective role: Reduction in Aβ plaques. Detrimental role: No reports.
	PD	PD marmosets; patients with PD	Downregulated in substantia nigra following nigral cell degeneration; upregulated in serum and CSF	Protective role: Protects tyrosine hydroxylase positive cells against toxic. Detrimental role: *Opn* (^–/–^) mice showed less death of tyrosine hydroxylase positive cells and dopaminergic fibers.
	Stroke	Patients with stroke	Upregulated in plasma	Protective role: Reduced mean infarct volume, ameliorated neurological deficits, mediated anti-inflammatory effect. Detrimental role: No reports.
LCN2	AD	Patients with AD	Upregulated in plasma but downregulated in CSF	Increase in neuroinflammation; reduction in Aβ plaque clearance; reduction in dehydrogenase activity and survival of wild-type astrocytes.
	PD	PD mice; patients with PD	Upregulated in substantia nigra; negative corrected with dopaminergic neurons	Disruption of the nigrostriatal dopaminergic projections; Involved in the induction of abnormal locomotor behaviors.
	Stroke	Patients with stroke	Upregulated in plasma especially in the acute stage	Increase in BBB permeability, neurological deficits, cerebral infarction, and infiltration of neutrophils in the acute stage. Stimulated microglia and astrocytes into potentially pro-recovery phenotypes in the later stage.
	MD	Chronic stress mice	Upregulated in the hippocampus after stress	Reduction in dendritic spines and ability to regulate neuronal excitability.
BMSCs	AD	AD mice	**/**	Reduction in Aβ plaques and dystrophic neurites; Increase in neovascularization.
	PD	PD mice	**/**	Differentiation into functional dopaminergic neurons; reduction in microglial activation and tyrosine hydroxylase-positive neuronal loss.
	Stroke	Ischemic stroke rats	**/**	Promoted neurogenesis and angiogenesis, inhibited neuronal apoptosis.
	MD	Depression model mice	**/**	Increase in hippocampal neurogenesis.

**OCN, Osteocalcin; LCN2, lipocalin-2; OPN, osteopontin; BMSCs, bone mass-derived mesenchymal stem cells; AD, Alzheimer’s disease; PD, Parkinson’s disease; MD, Mood Disorders; CSF, cerebrospinal fluid; Aβ, β-amyloid; BBB, blood-brain barrier.**

### AD

AD, a progressive and neurodegenerative disease, is the most common cause of dementia. Initially, patients with AD often present with mild memory loss, which over time further deteriorates, resulting in the loss the of ability to live independently. The major pathologic characteristics of AD are neurofibrillary tangles and β-amyloid (Aβ) plaques (Hardy and Higgins, 1992; De Strooper and Karran, 2016). Though the pathogenesis of AD is still not clear, the excessive accumulation of Aβ plaques, which are generated by the abnormal processing of the amyloid precursor protein, is regarded as the cause of the deregulation of kinases and phosphatases that results in neuroinflammation and impairment of synaptic and neuronal function associated with the disease (Hardy and Higgins, 1992). A growing number of studies have supported that abnormal bone metabolism may participate in the pathogenesis of AD.

Clinical studies have suggested an association between circulating levels of undercarboxylated OCN and cognitive performance (Bradburn et al., 2016; Puig et al., 2016). OCN was also shown to be sufficient to rescue age-related cognitive decline and decrease anxiety-like behavior (Khrimian et al., 2017). Blood extracted from young mice, and subsequently transfused into older mice, was found to rejuvenate brain function, which indicated that young blood contained effective factors that exerted nerve repair functions (Villeda et al., 2014). Khrimian et al. (2017) reproduced this experiment and discovered that OCN was necessary for young blood to exert nerve repair function. Therefore, OCN was regarded as an anti-gerontic hormone and the lack of OCN in old blood may be related to the pathogenesis of AD. However, to date, an explicit role for OCN in AD is unclear and needs further investigation.

LCN2 has been found to be decreased in cerebrospinal fluid (CSF) but elevated in plasma of patients with mild cognitive impairment and AD. In particular, plasma LCN2 was found to be negatively corrected with clinical dementia rating scores and positively corrected with mini-mental status examination scores, and is therefore regarded as a potential marker to predict the progression from mild cognitive impairment to AD (Choi et al., 2011; Naude et al., 2012). LCN2 may also be involved in AD pathogenesis through several mechanisms. First, LCN2 contributes to the neuroinflammation leading to the pathogenesis of AD. The chronic and excessive immune response could induce the upregulation of LCN2 in the brain (Naude et al., 2012; Dong et al., 2013). The upregulation of LCN2 then promotes the migration of microglia, astrocytes, and neurons, which play an important role in neuroinflammation (Lee et al., 2011, 2012). Second, the upregulation of LCN2 might inhibit Aβ plaque clearance. Insulin signaling was reported to inhibit Aβ plaques and promote the transport of Aβ out of the brain (Farris et al., 2003; Talbot and Wang, 2014). Interestingly, LCN2 was demonstrated to reduce insulin sensitivity (Yan et al., 2007; Law et al., 2010). Furthermore, LCN2 might regulate the cellular response to Aβ plaques. Aβ stimulation was found to decrease dehydrogenase activity and survival of wild-type astrocytes, but these effects were negated in *Lcn2*^(–/–)^ mice (Mesquita et al., 2014). Taken together, the results indicate that LCN2 may participate in the regulation of AD pathogenesis by influencing neuroinflammation, insulin signaling, and cellular responses associated with Aβ plaques. In conclusion, these studies indicate that LCN2 involve in the onset and progression of AD; therefore, control of LCN2 expression or activity may be the therapeutic target for AD.

OPN levels were found to be significantly elevated in the plasma and CSF of patients with AD (Sun et al., 2013). Similarly, Comi et al. (2010) found that OPN levels were significantly higher in patients with early stage AD and were correlated with the Mini-Mental Status score, making it a possible predictor for early AD. Moreover, Wung et al. (2007) discovered a significant increase in OPN expression in the hippocampus of patients with AD and that OPN showed positive correlation with Aβ plaques and age. Recently, OPN was also shown to have potential therapeutic effects against AD. Glatiramer acetate immunization markedly inhibited Aβ plaques and preserved cognitive function in an AD mouse model through upregulation of OPN, which regulated immunological profiles and physiological functions of macrophages to resist pathogenic factors of AD (Rentsendorj et al., 2018). Finally, OPN is known to bind with CD44 to exert its anti-apoptotic functions, which could be utilized to serve as a potential molecular checkpoint to reduce neuronal degeneration associated with AD (Lin and Yang-Yen, 2001).

Many reports have indicated the potential of BMSCs in the treatment of AD. Using a mouse model of AD, Lee et al. (2009) found that BMSC intracerebral injection promoted the reduction of Aβ through activation of microglia. Another study showed that transplantation of BMSCs overexpressing vascular endothelial growth factor into the hippocampus of AD mice promoted Aβ clearance and neovascularization, which was accompanied by behavioral benefits and alleviation of cognitive dysfunctions. Similarly, Wu et al. (2017) showed that extracellular vesicles secreted by BMSCs were effective at improving cognitive function and reducing the Aβ deposition and number of dystrophic neurites in an APP/PS1 mouse model. Moreover, BMSCs were found to play an important role in the replenishment of neural lineages and neurogenesis in the brains of AD mice. In summary, BMSCs may treat AD via regulating angiogenesis, neurogenesis, immunomodulation and eliminating the Aβ deposition.

### PD

PD is one of the most common progressive neurodegenerative disorders, characterized by motor deficits such as slowness of movement, rigidity, tremor, and postural instability (Braak et al., 2003). Neurons, and in particular dopaminergic neurons, in the PD brain gradually break down and die, leading to decreased dopamine levels, which is the primary cause of the motor symptoms of PD (Magalingam et al., 2015). The neuropathological hallmark of PD is the formation of Lewy body inclusions enriched in filamentous forms of the synaptic protein alpha-synuclein (Angot and Brundin, 2009).

The circulating levels of OCN are significantly lower in midlife than in adolescence and falling OCN levels have been demonstrated to be one of the important factors associated with age-related cognitive decline (Liu et al., 2008; Bradburn et al., 2016; Obri et al., 2018). Furthermore, OCN binds to neurons to increase dopamine synthesis, the low levels of which are associated with motor symptoms (Oury et al., 2013). Recently, a study found that OCN level was lower in CSF of 6-hydroxydopamine-induced PD rats than that in sham rats, and administration of OCN could ameliorate behavioral dysfunctions associated with PD (Guo et al., 2018). Further research showed that OCN could reduce the loss of tyrosine hydroxylase in the nigrostriatal system and regulate astrocytic and microglial functions (Guo et al., 2018). Gpr158 is the only central receptor of OCN found in the brain, but whether OCN can exert its neuroprotective efficacy in a PD rat model by binding to Gpr158 is still unknown (Khrimian et al., 2017; Cetereisi et al., 2019).

Kim et al. (2016) showed that LCN2 expression was significantly elevated in the substantia nigra of PD patients, and there was a negative correlation between LCN2 levels and dopaminergic neurons. In animal experiments, LCN expression was found to increase in the substantia nigra following injection of 6-hydroxydopamine in the medial forebrain bundle, suggesting that increased LCN2 levels may be associated with PD pathogenesis. Beyond that, LCN2 upregulation was discovered in neurotoxin-treated mouse models of PD. The dopaminergic neurotoxin, 1-methyl-4-phenyl-1,2,3,6-tetrahydropyridine (MPTP), can cause clinical symptoms similar to PD and is commonly used to induce PD mouse models (Song et al., 2021). LCN2 expression, in an MTPT induced mouse model, was found to be significantly upregulated in the brain, especially in reactive astrocytes of the substantia nigra and striatum (Kim et al., 2016). This upregulation of LCN2 was regarded as a potential pathogenic mechanism of PD, involving disruption of the nigrostriatal dopaminergic projections and induction of abnormal locomotor behaviors through neurotoxic iron accumulation and neuroinflammation (Kim et al., 2016). More importantly, they also found that gene knockout of LCN2 ameliorated the aforementioned PD symptoms in mice (Kim et al., 2016). Therefore, the development of regulatory method for LCN2 or the inhibitors of LCN2-induced neurotoxicity and neuroinflammation may be helpful for the treatment of PD.

OPN protein expression was discovered to be significantly low in marmosets after treatment with MPTP, including in the dopaminergic neurons of the substantia nigra (Iczkiewicz et al., 2006). Similarly, the same study found low OPN expression in the postmortem brains of patients with atypical parkinsonism (Iczkiewicz et al., 2006). The low expression levels of OPN following nigral cell degeneration indicated that OPN might play a central role in dopaminergic neuron survival. Further studies showed that the N-terminal fragment of OPN (OPN-N) containing arginine-glycine-aspartic acid (RGD)-peptide, an OPN fragment generated by thrombin cleavage, could protect tyrosine hydroxylase positive cells in the rat substantia nigra and primary ventral mesencephalic cultures from damage induced by 6-hydroxydopamine, lipopolysaccharide, and MPTP through its interaction with integrins, which subsequently altered glial activation (Iczkiewicz et al., 2010; Broom et al., 2015). These studies suggest that OPN can exert neuroprotective functions in PD models. In contrast, Maetzler et al. (2007) found that the level of OPN was elevated in PD patients and its absence led to reduced neurodegeneration in the MPTP mouse model. Besides, after MPTP treatment, OPN knockout mice showed less death of hydroxylase positive cells and dopaminergic fibers in the striatum compared with wild-type controls (Maetzler et al., 2007). The detrimental role of OPN in PD can be attributed to the inflammatory response. C-terminal fragment of OPN (OPN-C), another OPN fragment generated by thrombin cleavage, may be the main driver for inflammatory response. OPN-C was reported to interact with CD44 to inhibit IL-10 secretion and promote cell-cell adhesion (Weber et al., 1996). Collectively, these seemingly opposing roles of OPN in PD may be associated with the different functional domains of OPN or activation of different signaling pathways (Cappellano et al., 2021). Correctly understanding the molecular mechanism for the opposing roles of OPN will help to develop highly specific therapeutic tools via targeting OPN.

Growing evidence from experimental PD research has demonstrated that BMSC-mediated amelioration of PD is multimechanistic. Shetty et al. (2009) demonstrated that BMSCs could efficiently transdifferentiate into functional dopaminergic neurons both *in vitro* and *in vivo*. They then transplanted undifferentiated BMSCs into animal PD models and found significant behavioral improvements (Shetty et al., 2009). BMSCs were also found to have the capacity to induce neuroblasts to migrate to lesioned brain areas and enhance neurogenesis. Park et al. (2012) demonstrated that tail vein BMSC administration to PD mice increased neurogenesis in the substantia nigra and subventricular zone and also led to the differentiation of neural precursor cells into dopaminergic neurons in the substantia nigra. Furthermore, BMSCs are thought to exert their neuroprotective efficacy on dopaminergic neurons via anti-inflammatory effects by inhibiting microglial activation. Kim et al. (2009) used LPS to induce inflammation in a rat model of PD. They found that BMSCs administration significantly decreased tyrosine hydroxylase positive neuronal loss, microglia activation, TNF-α mRNA expression, and iNOS and TNF-α secretion.

### Stroke

Stroke, which is the leading cause of death and the primary reason for adult disability, can be divided into ischemic stroke and hemorrhagic stroke. Ischemic stroke is the most common type of stroke and caused by a blood clot that blocks a blood vessel in the brain. Another type of stroke is hemorrhagic stroke which is caused by the rupture of cerebral arteries (Rothwell et al., 2004; Polivka et al., 2019). Below, we reviewed the roles of LCN2, OPN and BMSCs in stroke except for OCN which has not been reported.

Plasma level of LCN2 was found to be significantly elevated in patients with ischemic stroke, especially in the early stages, and to be a risk factor for unfavorable modified Rankin scale scores, the occurrence of post-stroke infections and cardiovascular mortality (Anwaar et al., 1998; Falke et al., 2000; Hochmeister et al., 2016). In mice models of ischemic stroke induced by transient middle cerebral artery occlusion (tMCAo), serum level of LCN2 was acutely induced after tMCAo and was reduced at 48–72h post-tMCAo (Jin et al., 2014; Wang et al., 2015). The early induction of LCN2 indicated that LCN2 can be used as an early blood biomarker for stroke. Further researches showed that LCN2 deficient mice with fewer symptoms of BBB permeability, neurological deficits, cerebral infarction, and infiltration of neutrophils after tMCAo (Jin et al., 2014; Wang et al., 2015). They also revealed LCN2 promoted neuroinflammation through the activation of neutrophil infiltration, microglia/astrocytes, and the induction of pro-inflammatory cytokines and chemokines (Bi et al., 2013; Jin et al., 2014; Wang et al., 2015). Therefore, inhibiting LCN2 might be a promising therapeutic strategy to reduce post-stroke inflammation. Interestingly, Xing et al. (2014) suggested that LCN2 expressed in injured neurons may be as a “help-me” signal that stimulated microglia to increase interleukin-10 (IL-10) production and enhance phagocytosis, and stimulated astrocytes to upregulate levels of BDNF, glial fibrillary acid protein and thrombospondin-1. These studies indicate that LCN2 might play different roles during the acute and later stages of stroke.

OPN has been regarded to have a clinical and pathophysiological relevance with stroke. Carbone et al. (2015) found that the serum level of OPN was positively associated with poor outcome at day 90 after ischemic stroke, while Jing et al. (2013) showed that the serum level of thrombin-cleaved OPN could discriminate patients with ischemic stroke from healthy controls and was negatively correlated with the clinical outcome at 12 months after hospital discharge. OPN was also proposed to regulate repair processes and to protect neurons in stroke. Meller et al. (2005) showed that intracerebral ventricular OPN administration to murine stroke models could significantly reduce infarct size after tMCAo. Besides, Doyle et al. (2008) discovered that the RGD-containing peptide fragment of OPN, which was a twofold more effective neuroprotectant than the full-length OPN, was the effective peptide for OPN to treat with stroke. Similarly, Jin et al. (2016) found that OPN failed to decrease infarct volumes in the focal cerebral ischemia rat model when the RGD was replaced by arginine-alanine-alanine. They also found that the direct binding between RGD-containing peptide fragment of OPN and αvβ3 integrin might be the mechanism for OPN to mediated anti-inflammatory effect (Jin et al., 2016). These results indicate that OPN can be used as a novel biomarker, predictor and therapeutic target for stroke.

Transplantation of BMSCs is regarded as a promising therapy for stroke. Several mechanisms of BMSCs treatment for stroke are as follows. BMSCs-induced angiogenesis was considered important for neurological recovery in stroke. Bao et al. (2011) found that BMSCs transplantation could promote angiogenesis in Ischemic brain areas through stimulating Notch signaling pathway. Besides, BMSCs were also found to promote neurogenesis, inhibit neuronal apoptosis, and improve behavioral and motor function in the ischemic stroke through increasing neurotrophic expression, and differentiating into neurons and oligodendrocytes (Jahromi et al., 2015; He et al., 2017, 2019).

## The Role of Bone-Derived Modulators in Mood Disorders

Mood disorders, such as mania, bipolar disorder, and depression, are heterogeneous conditions that are characterized by complex genetics, unclear pathophysiology, and variable symptomatology. A growing number of clinical investigations have shown that patients affected by mood disorders have a high incidence of bone metabolism abnormalities, which can lead to low bone mass and increased risk of fracture (Gale et al., 2012; Williams et al., 2013; Chen et al., 2016; Cheng et al., 2016). Meanwhile, alterations in bone-derived hormones can influence brain development, function, and behavior and regulate of neurotransmitter synthesis (Otto et al., 2020). Below, we outline the possible relationships between the bone-derived modulators and mood disorders.

First, significantly lower OCN levels have been found in depression patients (Aydin et al., 2011). Furthermore, OCN knockout adult mice were shown to exhibit a significant increase in anxiety-like behavior, including increased aversion to open spaces and light and deceased exploratory activity, all of which were reversed by the administration of OCN (Oury et al., 2013). Others found that OCN derived from bone was able to cross the BBB and modulate transcription factors in neurons of the VTA, DRN, MRN, and CA3 region, which increased serotonin, dopamine, and noradrenaline release and inhibited GABA release (Oury et al., 2013; Khrimian et al., 2017), resulting in a reduction in anxiety-like behavior^6,25^. These results indicate that OCN may be a promising treatment tool for depression.

Another bone-derived factor related to mood disorders is LCN2. The nervous system undergoes a variety of adaptive changes to maintain homeostasis after psychological stress (Roozendaal et al., 2009). However, disorders of the regulatory functions that maintain homeostasis may lead to affective disorders. LCN2 is regarded as an important regulatory factor in the nervous system’s response to reply stress. Mariusz and co-workers demonstrated that LCN2 was significantly upregulated in the mouse hippocampus after stress, which resulted in the loss of dendritic spines and regulation of neuronal excitability (as a result of reduced dendritic spine actin mobility), and increased anxiety (Pawlak et al., 2003; Adhikari et al., 2010; Mucha et al., 2011). Therefore, LCN2 may be a therapeutic target to combat anxiety induced by stress.

BMSCs may be also a promising strategy for mood disorders, as they promote the expression of neurotrophic factors and neurogenesis. However, studies regarding the efficacy BMSCs in animal models of mood disorders are still limited. It is known that impaired hippocampal neurogenesis is related to depression (Thomas et al., 2007). Munoz et al. (2005) demonstrated that BMSC implantation promoted hippocampal neurogenesis. Furthermore, using a mouse model of depression, Tfilin et al. (2010) found that BMSCs migrated to the CA1 and CA2 regions, and the ipsilateral dentate gyrus after intracerebroventricular injection, which improved depressive-like behavior and hippocampal neurogenesis (Tfilin et al., 2010).

## Potential Clinical Application of Bone-Derived Modulators in Neurological Disorders

Until now, patients with neurological disorders, the second main cause of death worldwide in 2016, still have no access to effective treatments (Feigin et al., 2019). Therefore, there is an urgent need to develop new and effective treatments for those suffering from neurological disorders. The crosstalk between bone and brain tissues provides a new perspective to research new therapeutic approaches for neurology.

Although, as mentioned above, a growing number of bone-derived molecules have been demonstrated to affect brain function, most of these discoveries are limited to preclinical studies except for BMSCs (Otto et al., 2020). A growing number of basic research and clinical trials regarding the therapeutic effects of BMSCs in neurological diseases have shown that BMSCs have great potential to improve patient symptoms and quality of life (Badyra et al., 2020). To date, more than 20 clinical trials have been registered on the ClinicalTrials.gov^1^. However, more research is needed to evaluate different doses, methods of administration, and cell culture conditions, among others, to obtain the best therapeutic regimen for patients.

Several researchers have demonstrated that young blood could reverse age-related impairments, including loss of cognitive function and synaptic plasticity (Villeda et al., 2011, 2014; Khrimian et al., 2017). These reports indicate that young blood may contain powerful molecules that exert rejuvenating effects in the brain, several of which may have direct clinical applications.

An unexpected and remarkable discovery indicated that bone-derived OCN was necessary for young blood to exert an anti-geronic function. Khrimian et al. (2017) showed that the administration of plasma from young *Ocn*^–/–^ mice to older mice did not improve anxiety-like behavior or cognitive function in several behavioral tests. However, improvements were found when wild-type control plasma was used. Importantly, no side effects were detected after plasma administration. Taken together with the other OCN functions presented in this review, we believe that OCN has prospective anti-geronic benefits with low side effects and high effectiveness. However, it is important to consider that the research results are limited to mice, and that future studies in humans are necessary.

## Conclusion and Future Perspectives

Since the discovery of the essential roles of bone-derived OCN in brain development and function, the number of studies regarding the regulation of the brain by bone has increased considerably. Studies have revealed that bone can influence anxiety, memory, acute stress response, and even appetite by acting on various brain regions (Oury et al., 2013). These studies reconfirm the classic principle of physiology that no single organ can develop alone (Qin et al., 2016). Future therapeutic strategies could be designed to target the crosstalk between the brain and bone.

The remarkable discoveries regarding the roles of bone-derived modulators in brain functions not only add to our knowledge of brain function-related factors but also provide revolutionary insights from the perspective of the pathophysiology of psychiatric and neurological diseases. However, the roles of bone-derived modulators in the brain are not fully known (Han et al., 2018). It has been shown that OCN and Gpr158 (OCN receptor) are not only expressed in the hippocampus and ventral tegmental area but also the motor areas, auditory and somatosensory areas of the cortex, piriform cortex, and retrosplenial area (Oury et al., 2013; Khrimian et al., 2017). However, it remains unclear whether OCN binds to Gpr158 in all of these areas to exert different functions.

LCN2 was formerly thought to be secreted exclusively by adipose tissue. However, recent research demonstrated that bone was the main organ where LCN2 was expressed (Mosialou et al., 2017). Though the role of LCN2 in the central nervous system has been extensively studied, there is little research related to the role of bone-derived LCN2 in the brain (Mucha et al., 2011; Ferreira et al., 2018). Importantly, the expression level of LCN2 in bone was at least 10-fold higher than that in adipose and other tissues, implying that bone-derived LCN2 might play the main effect in the brain (Mosialou et al., 2017).

OPN, as a double-edged sword, not only exerts a detrimental role but also exerts a protective role in neurological disorders (Cappellano et al., 2021). Therefore, it is important to study the role of OPN in the development of diseases of the central nervous system. In particular, further research is needed to determine the mechanisms involved in the opposing roles of OPN-mediated neuronal toxicity and OPN-mediated neuroprotection in the development of neurological disorders. Correctly understanding the mechanisms of these opposing roles of OPN will help to develop highly specific therapeutic tools via targeting OPN. Another interesting point is that OPN has been regarded as an injury and repair biomarker of PD in two different respective studies (Maetzler et al., 2007; Broom et al., 2015). However, contrasting findings may both be correct because of the opposing roles of OPN in PD. We speculate that low levels of OPN may inhibit wound healing and tissue injury through immune-mediated mechanisms, whereas high levels of OPN may induce excessive tissue injury.

As we mentioned above, BMSC treatment has been widely studied both in preclinical studies and clinical trials, and has great potential to treat neurological disorders (Badyra et al., 2020). However, many aspects need to be improved to obtain the best therapeutic regimen for patients. Thus far, several types of BMSC transplantation have been established, including intravenous, intra-arterial, intrathecal, intranasal, intraspinal, intracerebroventricular, and intracerebral. Yet, thus far, there has been no consensus which method/methods are best. Though intravenous is the most common and feasible way of transplantation in the clinic, many of the cells become trapped in the lungs, which limits the therapeutic outcome of BMSCs (Agadi and Shetty, 2015). Research shows that invasive methods of transplantation may be more effective when BMSCs are transplanted directly into the injured brain (Jarocha et al., 2015). However, these invasive methods may induce more adverse reactions. Therefore, it is important to identify the best BMSC transplantation methods that are closely related to therapeutic outcome (Ben-Shaanan et al., 2008). Other aspects, including medication time and dosage, also need to be optimized in clinical trials to realize the best therapeutic regimen for patients.

Sclerostin, an osteocyte-specific glycoprotein encoded by the SOST gene, which can elevate bone resorption and reduce bone formation by inhibiting the Wnt/β-catenin pathway. Recently, several studies have shown that the Wnt/β-catenin pathway plays an important in neurogenesis, synaptic plasticity, neuronal survival, and BBB integrity (Baron and Kneissel, 2013; Williams, 2014; Shah et al., 2015; Noelanders and Vleminckx, 2017; Koide and Kobayashi, 2019). This leads us to question whether sclerostin crosses the BBB and influences brain functions via the Wnt/β-catenin pathway. Further investigations are needed to answer this and other unknowns regarding bone-derived modulators.

In the future, it will be necessary to decode the detailed regulatory network between bone and brain function. We believe that more bone-derived modulators will be identified that directly influence brain function and that these modulators could serve as potential molecular targets for the treatment of neurological disorders.

## Author Contributions

CL designed and supervised the manuscript. HC consulted the literature and wrote the manuscript. YW and DS provided advice on the manuscript. All authors read and agreed to publish the manuscript.

## Conflict of Interest

The authors declare that the research was conducted in the absence of any commercial or financial relationships that could be construed as a potential conflict of interest.

## Footnotes

1 https://www.clinicaltrials.gov/
